# Coordinated Implementation of Chikungunya Virus Reverse Transcription–PCR

**DOI:** 10.3201/eid1503.081104

**Published:** 2009-03

**Authors:** Marcus Panning, Remi N. Charrel, Oliver D. Mantke, Olfert Landt, Matthias Niedrig, Christian Drosten

**Affiliations:** Bernhard Nocht Institute for Tropical Medicine, Hamburg, Germany (M. Panning); Université de la Méditerranée, Marseille, France (R.N. Charrel); Robert Koch Institute, Berlin, Germany (O.D. Mantke, M. Niedrig); TIB MOLBIOL, Berlin (O. Landt); University of Bonn Medical Centre, Bonn, Germany (C. Drosten)

**Keywords:** Chikungunya fever, real-time RT-PCR, implementation, external quality assessment, dispatch

## Abstract

A preformulated chikungunya virus real-time reverse transcription–PCR, quality-confirmed oligonucleotides, and noninfectious virus controls were distributed by the European Network for the Diagnosis of Imported Viral Diseases. An international proficiency study with 31 participants demonstrated that ad hoc implementation of molecular diagnostics was feasible and successful.

Chikungunya fever, caused by chikungunya virus (CHIKV), is an acute febrile illness that causes severe and long-lasting arthralgia (*1*). A recent and ongoing epidemic in the Indian Ocean area extended far beyond this region and caused hundreds of imported cases worldwide (*2*–*4*). Chikungunya fever is difficult to clinically distinguish from co-endemic diseases such as malaria or dengue fever. Laboratory testing is required for appropriate case management and public health response (*5*). Pilot studies have shown that reverse transcription–PCR (RT-PCR) reliably detects acute infections in humans (*3*,*6*), but many laboratories were not ready to conduct such tests when this epidemic occurred.

During 2006 and 2007, the European Network for the Diagnosis of Imported Viral Diseases (ENIVD) received requests by many laboratories for assistance with CHIKV diagnostics. On the basis of experiences during the outbreak of severe acute respiratory syndrome (SARS) in 2003 (*7*), an ENIVD member laboratory distributed a then-unpublished real-time RT-PCR protocol that had been evaluated with a large number of clinical samples from imported cases to laboratories asking for assistance (*3*). To determine efficacy of RT-PCR testing for CHIKV, we distributed testing materials to 31 participating laboratories in an external quality assurance study. Laboratories sent their results to ENIVD for analysis of efficacy.

## The Study

Information distributed to laboratories asking for assistance with CHIKV RT-PCR included reaction chemistry setup, cycling profile, and primer and probe sequences. A quantified CHIKV in vitro RNA transcript containing 9 × 10^10^ subgenomic RNA copies/μL was used as a noninfectious positive control. Additional measures were taken to provide proper primers and probes because these components are most vulnerable to variation when assays are adapted from protocols, e.g., because of synthesis errors or poor purification. Primers and probes were synthesized in large reference lots and stored centrally at an oligonucleotide factory. Samples of these lots were validated by the reference laboratory and confirmed to provide full sensitivity as achieved with the original primers used in developing the prototype assay (*3*). Recipients of protocols were invited to order and use aliquots of primers directly from the validated reference lot.

To receive feedback on performance of this method and other methods of CHIKV detection, a proficiency study was organized among ENIVD members. All participants were informed about the option of obtaining the preformulated assay. Laboratories in Europe (22), Asia (6), South America (2), and Africa (1) participated.

Inactivated and stable testing material was generated from cell culture supernatants of 4 CHIKV strains from the epidemic in the Indian Ocean area (1 each from Seychelles, Mauritius, Réunion Island, and India) and 1 East/Central Africa strain (S27). Virus solutions were inactivated by heating at 56°C for 1 h and gamma irradiation with 30 kGy. Residual infectivity was excluded by 3 blind passages of a sample of each solution on Vero cells. Solutions were diluted in human fresh-frozen plasma, aliquoted (100 μL), and lyophilized. Test aliquots were reconstituted in 100 μL of water, and CHIKV RNA was quantified by RT-PCR (*3*). Lyophilized samples were shipped at ambient temperature to participating laboratories. Each shipment contained a coded panel of 9 CHIKV RNA positive– and 3 CHIKV RNA–negative lyophilized samples with virus concentrations shown in Table 1. Participants were asked to test the material with any molecular assay routinely used for detecting CHIKV in human plasma or with the preformulated test. We requested test results and assay details (PCR formulations and extraction methods). A total of 36 sets of results were received by the study coordinator, including 3 double sets from 3 laboratories that used 2 methods each. One laboratory provided triple sets of results from 3 tests.

**Table 1:** Positive samples in external quality assessment panel for detection of CHIKV by reverse transcription–PCR*

Sample code	Origin of strain	Virus RNA concentration, copies/mL	Laboratories with positive detection, %
CHIK #2	Réunion Island	10,487,171	100
CHIK #9	Réunion Island	745,257	77.4
CHIK #4	Réunion Island	86,197	83.9
CHIK #12	Réunion Island	7,040	48.4
CHIK #5	Réunion Island	1,076	22.6
CHIK #6	India	918,259	96.8
CHIK #10	Seychelles	526,268	87.1
CHIK #1	Mauritius	564,192	83.9
CHIK #11	East Africa	1,131,422	87.1

*CHIKV, chikungunya virus.

We used 2 criteria to define successful participation in the external quality assessment study. First, those samples containing >7,040 RNA copies/mL should be correctly identified. Analogous to previous external quality assessments (*8*–*11*), we chose this threshold because it is ≈5–10× above the limit of detection of current CHIKV RT-PCR protocols (*3*,*12*). Second, no false-positive results were allowed in virus-free samples.

Samples containing 10,487,171 RNA copies/mL were correctly detected by all participating laboratories (Table 1). Fifteen (48%) of the laboratories were able to detect samples containing >7,040 RNA copies/mL. Only 22.6% correctly detected the sample with 1,076 copies/mL. Of 31 laboratories, 14 (45.2%) met all proficiency criteria. Seventeen laboratories missed the proficiency criteria because of a lack of sensitivity. Two of these laboratories reported >1 false-positive result. Both laboratories had used a nested RT-PCR, which likely indicated cross-contamination during RT-PCR procedures. No other laboratories reported false-positive results.

To project performance of a hypothetical average laboratory, cumulative fractions of positive results reported for each test sample were correlated against RNA concentrations in samples and subjected to probit analysis. This procedure used a dose-response model, which predicted for the average laboratory that a 50% certainty of detection was achieved for CHIKV plasma concentrations >10,000 RNA copies/mL (95% confidence interval [CI] 3,162–19,952 copies/mL) (Figure). A 95% certainty of detection was achieved for CHIKV plasma concentrations >7,943,282 copies/mL (95% CI 2,511,886–39,810,717 copies/mL).

**Figure:**
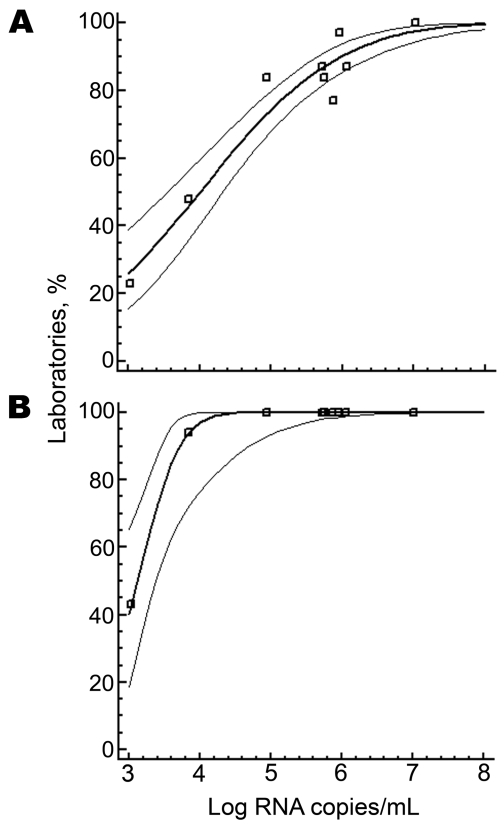
Probit analysis of laboratories with a positive result (y axes) for chikungunya virus in relation to viral RNA concentration in positive samples (x axes). A) Laboratories using in-house reverse transcription–PCRs (RT-PCRs) (n = 18) had a 50% certainty of having a positive result at 10,000 RNA copies/mL (95% confidence interval [CI] 3,162–19,952). B) Laboratories using a preformulated RT-PCR (n = 13) had a 50% certainty of having a positive result at 1,288 RNA copies/mL (95% CI 416–2,344). Data points represent individual samples in the test panel. Thick line is the regression line calculated on the basis of a probit model (dose-response curve), and thin lines are 95% CIs. Data fit into the model with p<0.00001.

To evaluate critical criteria in laboratory practice, we determined whether particular components of laboratory procedures had any systematic influence on laboratory performance. Selection of criteria was based on experiences from earlier external quality assessment studies (*8*,*9*,*11*). We evaluated automated versus manual RNA extraction methods, 1 widely distributed procedure for RNA extraction (viral RNA mini kit; QIAGEN, Hilden, Germany), any real-time RT-PCR, any nested RT-PCR, or the preformulated RT-PCR distributed with this study. Cumulative fractional positive results of all low- and medium-concentration samples (<86,197 copies/mL) were subjected to multifactor analysis of variance, which eliminated influence of other defined factors in each analysis. The only technical factor that increased sensitivity was the preformulated RT-PCR (Table 2). Thirteen (42%) of 31 participants used this assay. Another factor with nonsignificant benefit (p = 0.08) was use of automated RNA extraction.

**Table 2:** Possible technical factors influencing performance of laboratories in detection of CHIKV*

Factor	No. laboratories	p value for positive influence on sensitivity
QIAGEN† viral RNA extraction kit	23	0.2
Any automated RNA extraction procedure	8	0.08
Preformulated CHIKV real-time RT-PCR protocol	13	0.03
Any real-time CHIKV RT-PCR	27	0.3
Any nested CHIKV RT-PCR	6	0.37

*CHIKV, chikungunya virus; RT-PCR, reverse transcription–PCR. †Hilden, Germany.

## Conclusions

Because of little disease activity before the epidemic, laboratories inside and outside epidemic regions were not prepared to detect CHIKV when the epidemic occurred. In a similar situation during the SARS epidemic in 2003, we demonstrated that rapid provision of a commercial test kit could greatly assist laboratories worldwide, enabling them to perform state-of-the art molecular diagnostics during the epidemic (*7*,*9*). However, for chikungunya fever, commercial firms did not rapidly prioritize development of CHIKV test kits. ENIVD attempted to assist implementation of molecular diagnostics on an ad hoc basis by distributing a validated CHIKV RT-PCR and all required reagents.

Our proficiency study showed surprisingly good overall performance of participating laboratories than most of our previous external quality assessments (*8*,*10*). Analysis of factors identified that this success was primarily due to the preformulated assay. In our earlier external quality assessments on detection of emerging viruses, many participants used diagnostic methods reported in the literature, which did not provide technical features such as real-time PCR (*8*,*9*,*11*). The assay distributed in this study was technically advanced, and its efficient adaptation was supported by providing quality-controlled oligonucleotides and controls. This in-house assay was readily implemented by a large number of laboratories. It improved diagnostic proficiency similar to the commercial assay distributed during the SARS epidemic (*9*). We showed that novel PCR diagnostics for emerging diseases can be implemented on an international scale. However, enhanced support by reference laboratories through efficient collaborative networks of laboratories is indispensable. Public health organizations should be encouraged by these data to strengthen and extend networking between diagnostic laboratory facilities.
