# Erratum: Vol. 15 No. 3

**DOI:** 10.3201/eid1508.999000

**Published:** 2009-08

The name of Oliver Donoso Mantke was incorrect in the author list for the article Coordinated Implementation of Chikungunya Virus Reverse Transcription–PCR (M. Panning et al.). The article has been corrected online (www.cdc.gov/eid/content/15/3/469.htm).

